# Mesoporous Matrices as a Promising New Generation of Carriers for Multipolymorphic Active Pharmaceutical Ingredient Aripiprazole

**DOI:** 10.1021/acs.molpharmaceut.3c00524

**Published:** 2023-09-27

**Authors:** Aldona Minecka, Magdalena Tarnacka, Karolina Jurkiewicz, Daniel Żakowiecki, Kamil Kamiński, Ewa Kamińska

**Affiliations:** † Department of Pharmacognosy and Phytochemistry, Faculty of Pharmaceutical Sciences in Sosnowiec, Medical University of Silesia in Katowice, 41-200 Sosnowiec, Poland; ‡ A. Chelkowski Institute of Physics, University of Silesia in Katowice, 41-500 Chorzow, Poland; § Chemische Fabrik Budenheim KG, Rheinstrasse 27, 55257 Budenheim, Germany

**Keywords:** aripiprazole, mesopores, polymorphic forms, dissolution rate, drug carrier, crystallization

## Abstract

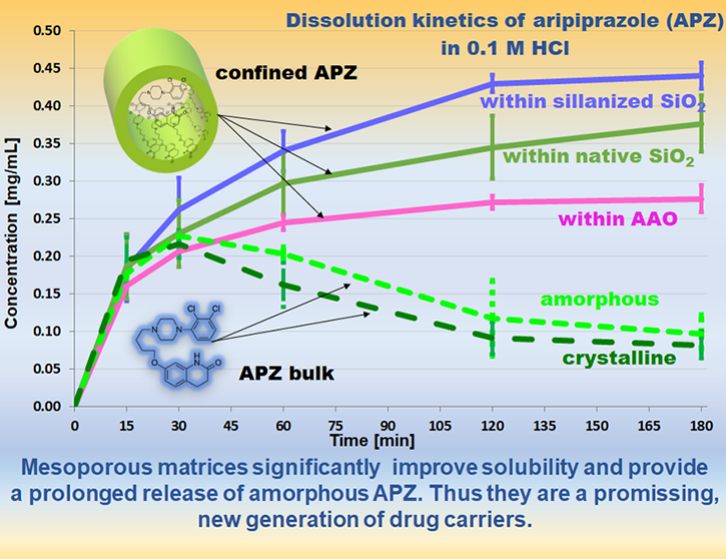

The enhancement of the properties (i.e., poor solubility and low bioavailability) of currently available active pharmaceutical ingredients (APIs) is one of the major goals of modern pharmaceutical sciences. Among different strategies, a novel and innovative route to reach this milestone seems to be the application of nanotechnology, especially the incorporation of APIs into porous membranes composed of pores of nanometric size and made of nontoxic materials. Therefore, in this work, taking the antipsychotic API aripiprazole (APZ) infiltrated into various types of mesoporous matrices (anodic aluminum oxide, native, and silanized silica) characterized by similar pore diameters (*d* = 8–10 nm) as an example, we showed the advantage of incorporated systems in comparison to the bulk substance considering the crystallization kinetics, molecular dynamics, and physical stability. Calorimetric investigations supported by the temperature-dependent X-ray diffraction measurements revealed that in the bulk system the recrystallization of polymorph III, which next is converted to the mixture of forms IV and I, is visible, while in the case of confined samples polymorphic forms I and III of APZ are produced upon heating of the molten API with different rates. Importantly, the two-step crystallization observed in thermograms obtained for the API infiltrated into native silica templates may suggest crystal formation by the interfacial and core molecules. Furthermore, dielectric studies enabled us to conclude that there is no trace of crystallization of spatially restricted API during one month of storage at *T* = 298 K. Finally, we found that in contrast to the crystalline and amorphous bulk samples, all examined confined systems show a logarithmic increase in API dissolution over time (very close to a prolonged release effect) without any sign of precipitation. Our data demonstrated that mesoporous matrices appear to be interesting candidates as carriers for unstable amorphous APIs, like APZ. In addition to protecting them against crystallization, they can provide the desired prolonged release effect, which may increase the drug concentration in the blood (resulting in higher bioavailability). We believe that the “nanostructirization” in terms of the application of porous membranes as a novel generation of drug carriers might open unique perspectives in the further development of drugs characterized by prolonged release.

## Introduction

It is well-known that the bioavailability of most pharmaceuticals available today in the market is determined by the solubility of their active pharmaceutical ingredients (APIs). All widely known formulation procedures, such as micronization,^1^ cocrystallization,^2^ granulation,^3^ salt formation,^4^ prodrug design,^5^ amorphization,^6,7^ etc., are intended to improve this parameter.^8^ The selection of the most appropriate method depends on physiological factors (site of action, administration route, pH of body fluids) as well as the physicochemical characteristics of a given API, e.g., its chemical structure, susceptibility to thermal degradation, reactivity, or tendency to form various polymorphic forms. The last phenomenon is very common among many pharmaceuticals, e.g., paracetamol (acetaminophen),^9^ ritonavir,^10^ some local anesthetic compounds (lidocaine, procaine, benzocaine),^11^ statins,^12^ alendronic acid,^13^ rifaximin,^14^ or aripiprazole.^15,16^ In light of current knowledge, different polymorphic forms of the same chemical compound may vary in their physicochemical properties, such as melting and sublimation temperatures, density, hardness, vapor pressure, refractive index, color, crystal shape, stability, hygroscopicity, reactivity, mechanical properties, solubility, as well as heat and rate of dissolution, which affect the bioavailability, and effectiveness of formulation procedures like the tableting.^17^ In this context, APIs’ polymorphism can have either positive or negative implications. The risk of uncontrolled polymorphic transformation from the stable, highly soluble state to the labile and poorly soluble one may lead to reduced drug efficacy, adverse side effects, or decreased stability of the drug formulation.^17^ On the other hand, a detailed investigation of pharmaceutical polymorphs and the factors determining their formation makes it possible to avoid the above-mentioned problems and develop formulations with better pharmacodynamic and pharmacokinetic properties to the market. Among the methods dedicated to seeking new polymorphic forms, one can mention the crystallization from suitably composed solutions, cocrystallization, and recrystallization from an amorphous state (bulk sample).^18,19^ Alternative promising pathways can be the use of high pressure or the infiltration of APIs into porous materials.

The potential of the latter is not yet fully explored. However, there is an increasing number of research, where porous systems are used as carriers for APIs, nucleic acids, and fluorescent or magnetic particles.^20−22^ One of the most popular materials from this group are spherical, mesoporous silica nanoparticles (MSN), built up from safe and inert inorganic siloxane^23^ with various morphology and pore shapes, e.g., MCM-41, SBA-15, Syloid XDP 3050, Syloid 244 FP, Aeroperl 300, and Neusilin US2.^24−28^ As shown in many reports, these materials play a crucial role in the stabilization of amorphous pharmaceuticals, i.e., the spatial restriction forced by pore diameter provides protection against crystallization.^29^ Besides MSN, other inorganic matrices with cylindrical pore shapes attract the attention of scientists due to both the possibility of the stabilization of amorphous APIs and controlling their recrystallization toward obtaining desired/even new polymorphic forms with well-defined properties. As an example, one can mention salol,^30,31^ fenofibrate,^32^ probucol,^33^ and cilnidipine,^34^ for which incorporation into anodic aluminum oxide (AAO) or silica (SiO_2_) membranes results in inhibition/slowing down (when compared to the bulk sample)/or even complete suppression of recrystallization from the amorphous phase. Importantly, for salol, depending on the pore size (diameter, *d*), stable (orthorhombic) or unstable (monoclinic) polymorphic forms of this API were obtained for *d* = 150 and 100 nm, respectively. Slightly different behavior was detected in probucol (PRO), where among two polymorphic forms of API, stable I and unstable II, the latter prevailed under confinement. In turn, for fenofibrate, recrystallization to the preferred form I, responsible for the therapeutic effect, was observed in nanospatial restrictions. Interestingly, in the bulk sample, also some amount of the metastable and not desired polymorphic form II was produced.

Furthermore, the application of porous materials often provides a modulated release effect. According to the pharmacokinetic studies, this effect also influences positively the bioavailability of pharmaceuticals (in addition to the already discussed solubility properties).^8^ In the simplest words, it ensures that the desired active compound concentration is maintained over time in the blood (or other body fluids). Thus, the modulated release helps to avoid sudden increases and decreases in the concentration of API in the system (especially immediately after drug administration), which can result in side effects, toxicity, or short-term action (forcing frequent, inconvenient administration of the drug). The most desirable effect is a slow, close to logarithmic increase in API concentration until it reaches a stable maximum that lasts as long as possible (the so-called prolonged release). Nevertheless, other release patterns, e.g., pulsatile in combination with prolonged, can also be designed depending on the expected therapeutic effect. Achieving this objective is possible because of many advanced drug delivery systems (DDS), such as specialized multichambered tablets e.g., with Oral Osmotic System (OROS) technology, hydrogels, micro- and nanospheres, liposomes, micelles, dendrimers, drug–polymer conjugates (like polymeric nanoparticles), nanotubes, specialized scaffolds, and described above mesoporous matrices.^35−37^ In the context of the latter ones, the effect of prolonged release and improvement of the dissolution rate was noted for naproxen,^38−40^ menthol,^41^ and celecoxib^42^ loaded into mentioned silica mesopores. In addition to the impact of amorphization, which results in higher solubility, both above-mentioned effects were assigned to the modified diffusion pathway determined by the pore shape, diameter, as well as its surface. However, still more research is needed to better understand this phenomenon.

In this paper, we investigate the molecular dynamics, crystallization tendency, long-term stability, and dissolution kinetics of the amorphous antipsychotic API aripiprazole (APZ) in bulk and infiltrated into anodic aluminum oxide (AAO) and two types of silica (SiO_2_) mesoporous membranes (native/untreated and silanized/treated) with similar pore diameters: *d* = 10 and 8 nm, respectively. The main purpose of our studies is to show the advantage of porous membranes as drug carriers that will provide better stability and enhanced solubility and hence bioavailability of the examined active substance.

## Materials and Methods

### Materials

**Figure 1:**
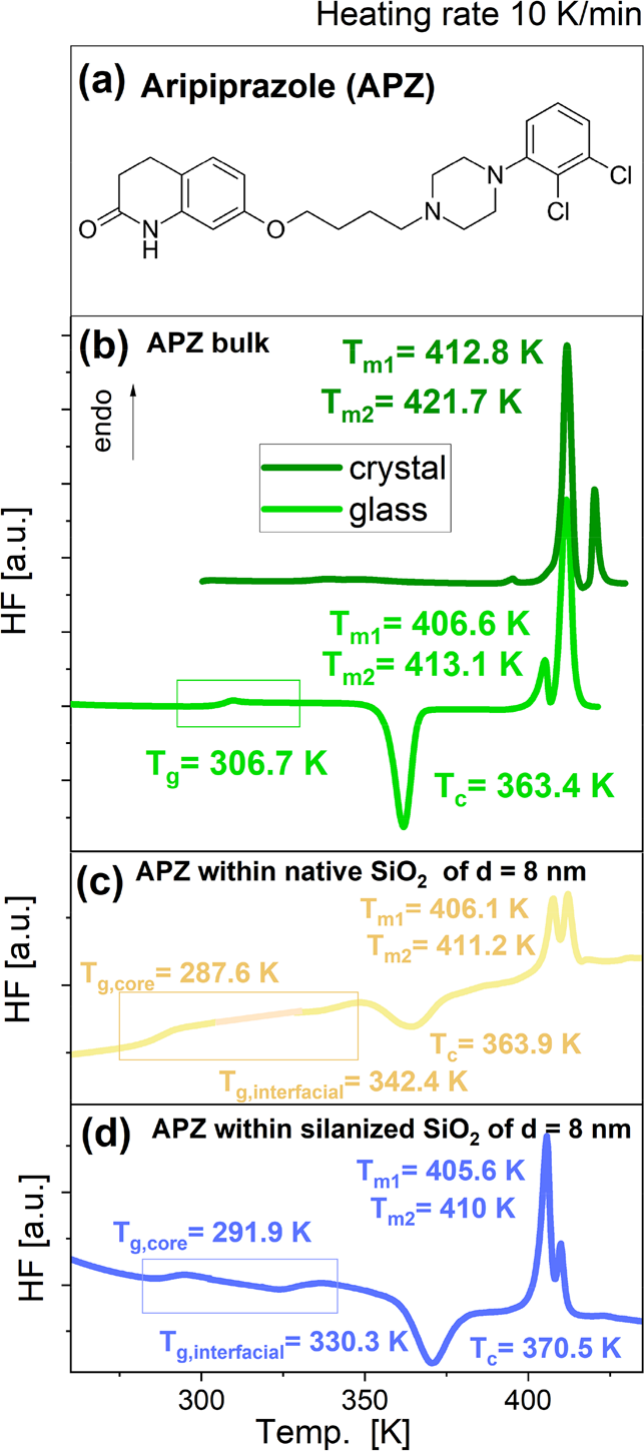
Chemical structure of the examined substance (a). DSC thermograms obtained for the bulk APZ (b) as well as the sample infiltrated into native (c) and silanized (d) silica templates of the same pore diameter, *d* = 8 nm, recorded at a heating rate of 10 K/min. *T*_c_ and *T*_m_ were determined from the maximum of the exothermic and endothermic peaks, respectively. *T*_g_ was obtained as the midpoint of the heat capacity increment.

Aripiprazole (APZ, C_23_H_27_Cl_2_N_3_O_2_, 7-{4-[4-(2,3-dichlorophenyl)piperazin-1-yl]butoxy}-3,4-dihydroquinolin-2(1*H*)-one, purity > 98%, *M*_w_ = 448.39 g/mol) was purchased from Sigma-Aldrich. Its chemical structure is presented in Figure 1(a). AAO membranes used in this study (with well-defined *d* = 10 nm) have been supplied by InRedox (details about the characteristics of these membranes are available on the Producer’s webpage.^43^ In turn, the native and silanized silica membranes (*d* = 8 nm) were prepared by electrochemical etching of silicon wafers and subsequent thermal oxidation.^44,45^ Applied procedures, including silanization, were also described in the Supporting Information to ref (46). It should be mentioned that in the case of silanized pores, the replacement of silanol groups by trimethylsilyl groups, which is a very effective process (degree of silanization ∼95%) results in a more hydrophobic surface and consequently weaker specific (H-bonding) interactions with the API molecules.^45^

### Samples Preparation

The amorphous bulk sample was obtained by vitrification. In turn, the path for preparing the infiltrated samples was a bit more complex. All three types of membranes were filled with the melted API by using infiltration forces inside the nanochannels. A detailed step-by-step procedure of the confined samples’ preparation has been described in the Supporting Information (SI). Herein, it should be stressed that the filling degree in all three cases (AAO, native, and silanized silica membranes) reaches ∼90%, and it was calculated considering the porosity of membranes and an assumption that the density of the infiltrated material does not change along the pore radius, as well as the shape of the pore is cylindrical. Although the value of this parameter depends on the porosity of the applied membranes, in our case, obtained values were very close to each other due to almost the same pore sizes of the used materials (8 nm and 10 nm).

### Differential Scanning Calorimetry (DSC)

Calorimetric measurements were carried out using a Mettler-Toledo DSC apparatus (Mettler-Toledo International, Inc., Greifensee, Switzerland) equipped with a liquid nitrogen cooling accessory and an HSS8 ceramic sensor. Temperature and enthalpy were calibrated by using indium and zinc standards. Samples were prepared in sealed aluminum crucibles (40 μL). In the case of confined systems, the membranes were crushed before being placed in the crucible. The crystalline bulk sample was scanned above its melting temperature (420–423 K), quenched, and scanned well above the melting point again (10 K/min). Confined samples were scanned over a temperature range of 260–440 K (10 K/min). Additionally, nonisothermal calorimetric measurements were carried out for all systems over the temperature ranges described above. In the case of the bulk sample, these experiments were made at a heating rate (ϕ) from 25 to 2.5 K/min. AAO membranes filled with APZ were scanned at ϕ from 20 to 7.5 K/min. In turn, both silica (native and silanized) systems were scanned atϕ from 20 to 2.5 K/min and from 17.5 to 5 K/min, respectively.

### X-ray Diffraction (XRD)

The temperature-dependent XRD studies of powdered samples (bulk APZ and SiO_2_ membrane-infiltrated samples) were performed with a Malvern Panalytical Empyrean diffractometer (Malvern Panalytical Ltd., Malvern, United Kingdom) using a nickel filtered Cu Kα_1,2_ source (λ = 1.5406 Å) and equipped with a PIXcell^3D^ ultrafast solid-state hybrid detector. An Anton Paar TTK 450 temperature chamber (Anton Paar GmbH, Graz, Austria) was used for temperature control. Measurements were carried out in the range of 303–423 K, in reflection mode in the Bragg–Brentano geometry, within the scattering angle 2θ range of 5–60°. The heating rate was around 0.3 K/min. In the case of APZ infiltrated into AAO, the XRD measurements were performed with a Rigaku Denki D-Max Rapid II diffractometer (Rigaku Corporation, Tokyo, Japan) in the Debye–Scherrer geometry using a monochromatized Ag Kα_1,2_ source (λ = 0.5608 Å). The heating rate was around 3 K/min.

### Broadband Dielectric Spectroscopy (BDS)

Complex dielectric permittivity (ε*(ω) = ε′(ω) – *i*ε″(ω)) measurements were carried out using the Novocontrol Alpha dielectric spectrometer (Novocontrol Technologies GmbH & Co. KG, Hundsangen, Germany) with a nitrogen gas cryostat and temperature controlled by a Quatro Cryosystem (better than 0.1 K) over the frequency range from 10^–2^ to 10^6^ Hz. Experiments for bulk APZ were performed after the vitrification process at ambient pressure in a broad range of temperatures (173–337 K). The samples were placed between two stainless-steel flat electrodes of the capacitor with a gap of 0.1 mm, mounted in a cryostat, and kept under a dry nitrogen gas flow during measurements.

Membranes (AAO, as well as native and silanized SiO_2_) filled with APZ were also placed in a similar capacitor (diameter: 10 mm, thickness of the membrane: 0.05 mm) and measured using two different protocols: (i) “slow-cooling” (S–C) from *T* = 343 K, where the sample has been slowly cooled (ϕ tends to 0 K/min) to *T* < *T*_g_, and (ii) “slow-heating” (S–H) of the quenched API, where the samples have been cooled below *T*_g_ (ϕ ∼ 100 K/min) and then slowly heated (ϕ tends to 0 K/min) to temperatures above 343 K. It should be mentioned that the confined samples are heterogeneous dielectrics, where the contribution from the used matrix and the API is considered. Collected dielectric spectra were recalculated accordingly to the approach presented in ref (47), because the equivalent circuit consists of two capacitors in parallel composed of ε*_APZ_ and ε*_AAO/SiO2_ (applied electric field is parallel to the long pore axes) and measured total impedance is related to the individual values through 1/Z*_sample_ = 1/Z*_APZ_ + 1/Z*_AAO/SiO2_. Additional isothermal, time-dependent (ca. 250 h) measurements were carried out at 305 K for APZ incorporated into AAO and SiO_2_ templates. After these experiments were completed, molecular dynamics of both samples were measured once again using a slow-heating procedure.

### Solubility Studies

In this study, the rate at which APZ dissolves in a 0.1 M hydrochloric acid solution (pH=1) was analyzed. For the powder samples (amorphous and crystalline APZ), approximately 10 mg of each was used for testing. In the case of API incorporated into mesoporous carriers (silanized SiO_2_, native SiO_2_, and AAO) the sample was crushed and an amount equivalent to about 10 mg of APZ was used for the analysis. The samples were placed in 20 mL of solvent and stirred with a magnetic stirrer at 500 rpm at ambient temperature for 180 min (with sampling points at 15, 30, 60, 120, and 180 min). Dissolved APZ was determined by the UV/vis method at a 248 nm detection wavelength using a T70 UV/vis Split-Beam Spectrophotometer equipped with flow-through quartz cuvettes with 1 mm path length (PharmaTest AG, Hainburg, Germany). Before measurement, the samples were filtered through a 0.45 μm Minisart-RC syringe filter (Sartorius, Goettingen, Germany), and diluted 20 times with 0.1 M hydrochloric acid.

## Results and Discussion

As a first step, we carried out calorimetric measurements (at heating rate = 10 K/min) of bulk APZ, and the sample incorporated into AAO, native, and silanized SiO_2_ templates to characterize thermal properties and phase transitions in these systems. Figure 1(b) presents thermograms obtained for crystalline (dark green line) and quenched/glassy API (light green line). As can be seen, during the heating of the crystalline sample, two endothermic peaks with maxima at *T* = 412.8 and 421.7 K are visible. To explain their origin, it should be stressed that APZ is the API with the high potential to create various polymorphic forms as well as hydrates, semihydrates, solvates, and cosolvates.^15,48−52^ Current reports indicate the existence of nine pure crystal forms (I–IX), which can be obtained by recrystallization, phase transitions, and spontaneous cocrystallization.^50^ Calorimetric characteristics of polymorphic forms I–V was presented by Braun et al.^48^ As shown by the authors, melting temperatures (*T*_m_ obtained as the onset of the peak) of particular polymorphs are equal to 421.5 K (for forms I and V; the last one was primarily called as X), as well as 416, 412, and 408 K (for forms II, III, and IV), respectively.^48,50^ Based on the above, one can conclude that both endothermic peaks in thermograms presented in Figure 1 may be associated with the melting of polymorphs III and I of APZ (respectively, at *T*_m1_ and *T*_m2_). However, it should be noted that *T*_m_s reported herein were determined as the peak maximum. Considering the intensity of these peaks, one can conclude that form III dominates in the initial crystalline sample. Interestingly, at around *T* = 397 K, one more small endothermic event is visible in the DSC curve of the crystalline API, but any of the known APZ polymorphs do not melt at this temperature. According to papers by Braun et al. and Britain, this event can be associated with the thermal transformation of form V (primarily designed by Braun as X) to form I.^48,50^ In turn, during heating of the quenched APZ, the heat capacity jump related to the glass transition phenomenon at *T*_g_ = 306.7 K is observed. Furthermore, at *T* = 363.4 K, an exothermic peak (indicating cold crystallization at *T*_c_), as well as two endothermic processes with maxima at *T* = 406.6 and *T* = 413.1 K, assigned to the melting of the recrystallized sample, can be detected. Considering again the data from refs (48) and (50), it can be supposed that the first endothermic event most probably indicates the presence of form IV (*T*_m1_), while the second one is form III (*T*_m2_). Our results obtained for the quenched APZ are in good agreement with DSC data published for this API by Knapik-Kowalczuk et al.,^53^ which showed similar values of *T*_g_ (306 K), *T*_c_ (363 K), and *T*_m_ (∼406 and ∼412 K). In addition, nearly the same melting temperatures (*T*_m_ = 405.7 and 411.4 K, assigned, respectively, to the IV and III polymorphs of APZ) were determined from calorimetric measurements carried out by Osiecka-Drewniak et al.^16^

DSC curves collected for APZ infiltrated into three types of templates differ from those measured for the quenched (bulk) sample since they revealed the presence of the two well-visible glass transition events located below and above *T*_g_ of the bulk API (see Figure 1(c,d)). According to the “two-layer”( or "core-shell") model,^54,55^ this phenomenon, known as a “double glass transition” (DGT), reflects the presence of two groups of molecules with different mobility: (i) “interfacial”, interacting directly with the inner surface of the pore wall and characterized by a higher *T*_g_ (so-called *T*_*g*_,_interfacial_), as well as (ii) “core”, located in the middle of nanochannels, which are characterized by bulk-like mobility and lower *T*_g_ (assigned as *T*_g,core_). The data obtained for the confined APZ are consistent with literature reports showing the occurrence of DGT for numerous low- and high-molecular substances under confinement,^54,56−58^ including APIs, such as salol,^30,31^ fenofibrate,^32^ probucol,^33^ and cilnidipine^34^ incorporated into AAO or native SiO_2_ membranes. Values of calorimetric *T*_g,core,_ and *T*_g,interfacial_ (measurements at 10 K/min) determined for APZ infiltrated into various types of matrices are presented in Figure 1(c,d) and Tables S2–S4. Interestingly, the difference between both *T*_g_s is higher for the API confined within native SiO_2_ membranes than in the case of APZ infiltrated into silanized SiO_2_ and AAO templates. This peculiar behavior might be a manifestation of various host–guest interactions in the investigated systems. In the context of silanized and native silica pores, the observed discrepancies (lower *T*_g,interfacial_ in the case of modified ones) might result from a lower density of silanol groups in the former system relative to the latter, which is due to the high degree (∼95%) of silanization, i.e., the introduction of hydrophobic trimethylsilyl groups to the structure. Consequently, the specific H-bonding interactions between the API and the matrix are weaker in the silanized silica templates.

**Figure 2:**
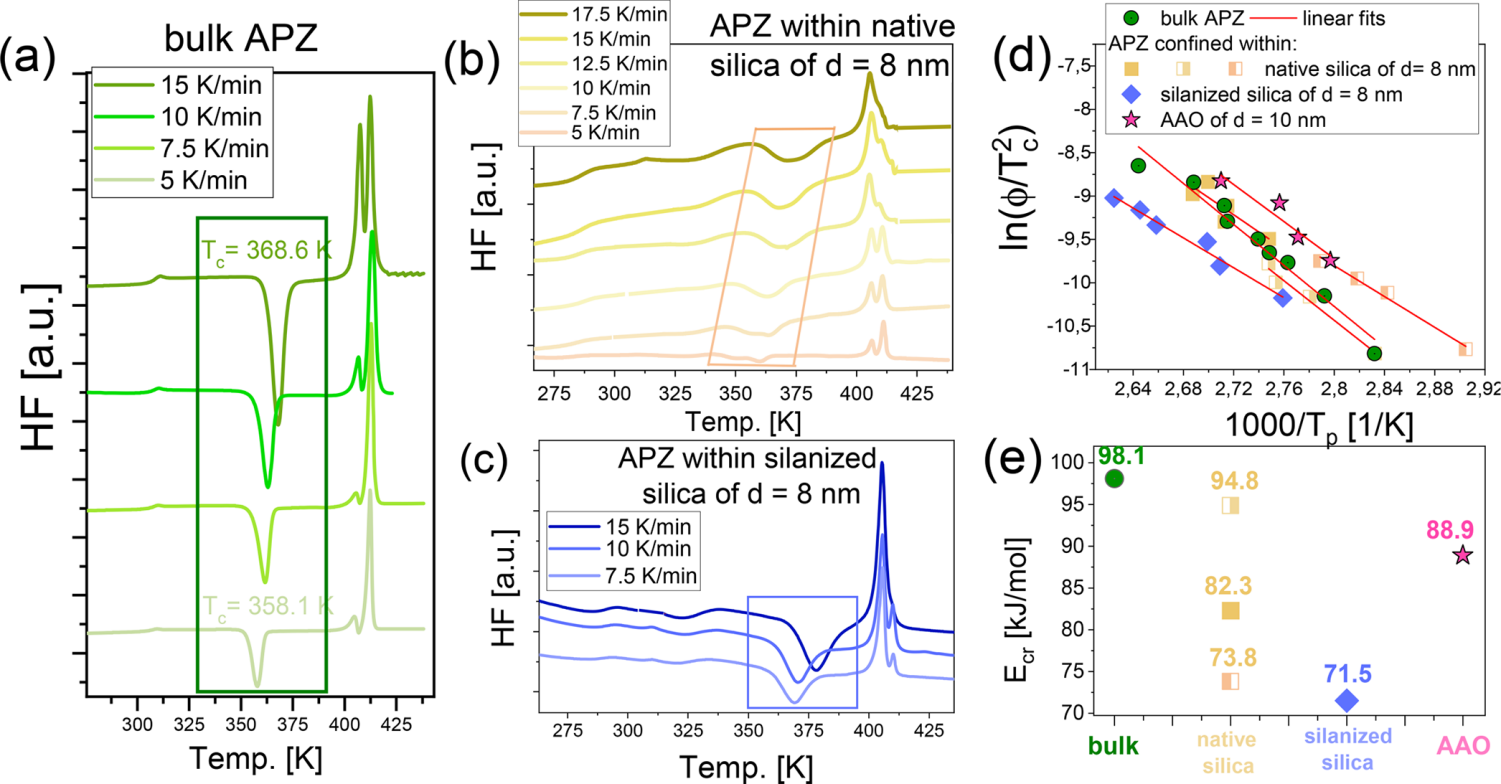
Representative DSC thermograms obtained for bulk APZ (a) together within those obtained for APZ confined within native (b) and silanized (c) silica templates of the same pore diameter, *d* = 8 nm, recorded at indicated heating rates. Kissinger plots for exothermic crystallization peaks in bulk APZ, APZ infiltrated into native silica, silanized silica, and alumina membranes (d) characterized by temperatures collected in Tables S1–S4. The solid lines indicate the linear fits to eq 1. The comparison of activation energies of nonisothermal crystallization estimated from the Kissinger approach (eq 1) for all examined systems (e).

It is important to note that as for the bulk sample thermograms registered for the confined APZ also indicate the cold crystallization peak near *T*_c_ = 360–370 K (for each membrane, the values of *T*_c_ are shown in Figure 1(c,d) and in Tables S2–S4). This is a quite interesting result indicating the high tendency of APZ to crystallize even in pores of very small diameter (*d* = 8 nm) irrespective of the template (silica vs alumina) or the character of the pore walls (native vs silanized). Having this result in mind, we decided to perform further nonisothermal systematic studies involving experiments with various heating rates (ϕ) to determine the activation barrier for the crystallization (*E*_cr_) for bulk APZ, as well as all confined samples. Representative data from these experiments are presented in Figure 2(a–c). As can be seen, thermograms recorded for bulk APZ and the sample infiltrated into native and silanized silica show that basically, at each (from 5 to 25 K/min), the glass transition (bulk) or DGT (confined samples) phenomenon, as well as the crystallization and two melting peaks, are observed. The temperatures of the glass transition, crystallization, and melting (*T*_g_s, *T*_c_s, *T*_m1_s, and *T*_m2_s) determined from the analysis of the nonisothermal DSC curves are given in Tables S1–S4. Moreover, for all examined systems, the position of each particular event in thermograms, as well as the intensity of those assigned to crystallization and melting processes are dependent on the applied heating rate. As can be seen, for the bulk sample, the glass transition is the least sensitive to the change of ϕ; *T*_g_ remains relatively constant at various ϕ (*T*_g_ ∼ 306–307 K, see Figure 2(a) and Table S1). In the case of the confined APZ, both *T*_g_s (*T*_g,interfacial_, *T*_g,core_) increase with increasing ϕ (see Figure 2(b,c) and Tables S2–S4). In comparison to *T*_g,core_, the increase of *T*_g,interfacial_ with ϕ is greater. Thus, the difference between both *T*_g_s in each thermogram is also getting higher as the heating rate rises. The temperature of cold crystallization (*T*_c_), as well as the intensity of the corresponding exothermic peak, decreases with decreasing ϕ for both bulk and confined samples (see Figure 2(a–c) and Tables S1–S4). Note that the obtained thermograms also revealed some important differences in the crystallization pattern of API incorporated into native and silanized SiO_2_ templates. As far as the samples were heated at ϕ higher than 10 K/min, the crystallization process was observed as a single peak occurring over a broad range of temperatures. On the other hand, at lower heating rates (ϕ ∼ 7.5–2.5 K/min), in the former system, two well-separated jumps suggesting the double crystallization process were detected; see Figure 2(b) and Table S2. One can be assumed that this effect is related to either the crystallization of various polymorphic forms or the crystallization of the core and interfacial fraction of API incorporated into native SiO_2_ membranes. This issue will be discussed in the further part of this paper. It should also be noted that analogous experiments for APZ infiltrated into alumina pores provided data of much worse quality and did not allow verification of whether a similar scenario occurs also in this case.

Having at hand thermograms measured at different heating rates, the Kissinger equation was applied to calculate the activation barrier for the crystallization.^59^ This model relies on the analysis of the variation in the peak temperature of crystallization (*T*_c_):
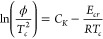
1were *C*_*K*_ is the fitting parameter. The outcomes of these analyses are presented in Figure 2(d), while the values of *E*_cr_ determined for each sample (bulk and confined) are shown in Figure 2(e). As mentioned before, since in the case of APZ confined within native SiO_2_ templates a double crystallization peak was observed in the thermograms measured at ϕ < 10 K/min, we analyzed both of them (as well as the one detected at ϕ > 10 K/min). As a result of that, we obtained three various Kissinger dependencies; see Figure 2(c). For a clearer illustration, these data are presented in a separate figure (Figure S1). Note that the values of *T*_c_ (maxima of exothermic peaks) were taken from Tables S1–S4. As can be seen, the activation barriers for API crystallization in the confined systems are lower than the *E*_cr_ calculated for the bulk sample (Figure 2(e)). Interestingly, the lowest value was determined for APZ within the silanized SiO_2_ pores. In turn, the crystallization energy calculated for API incorporated into native SiO_2_ membranes depends on the applied heating rate–for the data collected above and below 10 K/min; it varies from 74 to 95 kJ/mol. Obtained results indicating a drop of the activation barrier for the crystallization process occurring in pores seem to be quite nonintuitive. To explain this peculiar finding, one can suppose that most likely different polymorphs were formed in mesoporous templates or there is a strong contribution from the host–guest interactions to this phenomenon.

To gain insight into this issue, we took a closer look at the melting temperatures of the APZ crystallized in nanoconfinement and in the bulk sample. Interestingly, irrespectively on the porous membrane, the values of *T*_m_ (standard DSC measurements, ϕ = 10 K/min) are close to those obtained for the quenched bulk APZ (see Figure 1(b–d) and Tables S1–S4). Therefore, at first sight, one could suppose that polymorphs IV and III are formed in each confined API system.

Analyzing the thermograms obtained during heating the samples with different ϕ, one can conclude that the heat of fusion of a given polymorphic form varies with respect to the heating rate, nanospatial confinement, or the used template. The simplest situation is in the case of the data obtained for the bulk sample (Figure 2(a) and Table S1). In each nonisothermal DSC curve, two signals connected with melting are visible. As was described before for the quenched (bulk) sample heated with the rate of 10 K/min, *T*_m1_, and *T*_m2_ correspond probably to the melting of the IV and III polymorphic forms of APZ, respectively. The value of *T*_m2_ remains almost unchanged independently of the ϕ, while the value of *T*_m1_ slightly decreases with decreasing ϕ. Therefore, at lower ϕ, a little stronger separation of these two peaks can be noticed. Moreover, the intensity of the first melting signal gets lower with decreasing ϕ, which may suggest the reducing amount of polymorph IV in the sample. In turn, the position of the melting signals for APZ within native and silanized SiO_2_ pores is almost the same for various ϕ. Thus, *T*_m1_ and *T*_m2_ for these samples are also unchanged (405–406 K and 410–411 K, as well as ∼406 and ∼410 K, respectively, see Figure 2(b,c) and Tables S2 and S3). An analogous situation (very similar melting temperatures) was observed for the nonisothermal data obtained for APZ incorporated into AAO membranes (Table S4). Moreover, in contrast to the bulk sample, for both confined systems (APZ in native and silanized SiO_2_), only one melting peak, however, clearly broadened on the right side, was visible at the highest ϕ. Hence, one melting temperature could be determined (Figure 2(b,c), and Tables S2 and S3). For the API infiltrated within native SiO_2_ templates, the intensity of the second melting peak becomes greater with decreasing ϕ, with a simultaneous reducing intensity of the first melting signal. It may indicate that the amount of polymorphic form IV decreased, while the other form (III) increased with a lowering of ϕ. A slightly different scenario can be observed for APZ incorporated into silanized SiO_2_ pores (see Figure 2(c)). Here, the intensity of the first melting peak remains higher than the appearing second one regardless of the heating rate. It may suggest that the polymorphic form IV of APZ dominates in this system. In the case of AAO membranes, a slightly higher amount of form III with respect to polymorph IV was obtained at a given heating rate (data not shown).

**Figure 3:**
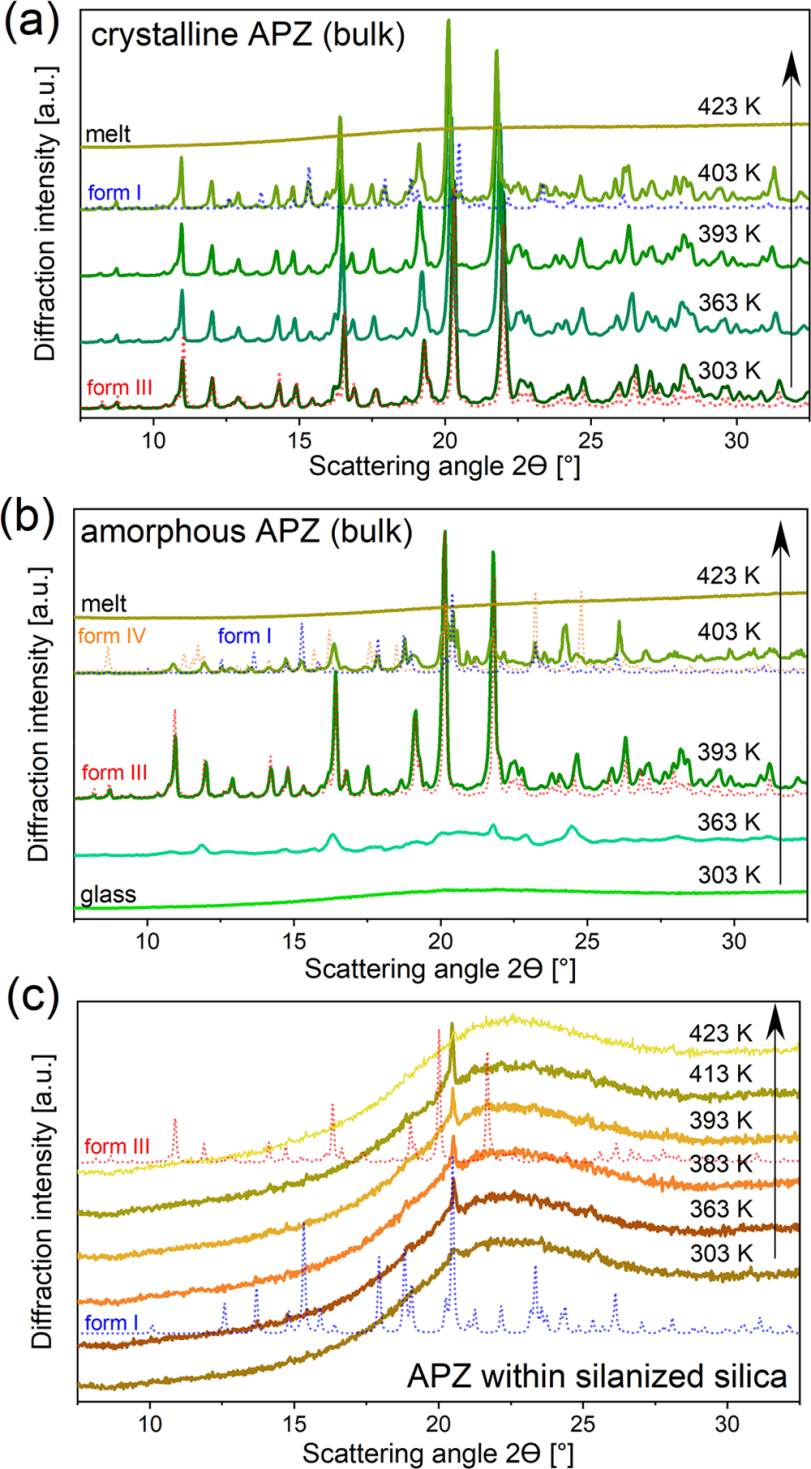
Evolution of XRD patterns on heating of aripiprazole: reference crystal (a), glass (b), and glass in silanized SiO_2_ pores (c).

Nevertheless, one has to be very careful with the assignment of endothermic peaks to a given polymorphic form for the sample that is nanospatially restricted. As presented in the literature, for such systems, very often a clear decrease in melting temperature is observed, which is in agreement with the Gibbs–Thomson relation linking the *T*_m_ with the size of crystallites^60−63^ To clarify this issue, X-ray diffraction studies for the reference crystalline APZ, its amorphous (glassy) form, and the API infiltrated into native, silanized SiO_2_ and AAO pores were performed. The results of temperature-dependent XRD measurements are presented in Figures 3 and S2. Unfortunately, the data for APZ in the native SiO_2_ templates are not shown due to a very weak signal from the API, which made it impossible to distinguish between polymorphic forms.

The diffraction data taken from the Cambridge Structural Database (CCDC 690583, form I; CCDC 690585, form III; and CCDC 690586, form IV) were presented in Figure 3 (dotted lines), enabling a quick recognition of the crystallizing polymorphs of APZ in the studied systems. It can be seen that starting APZ crystal was pure polymorphic form III, according to the nomenclature applied by Braun et al.^48^ On heating, at *T* > 400 K, a partial transformation of form III to form I took place, and further heating above *T* > 420 K led to the complete melting of both components. These findings are consistent with the analysis based on the DSC. In the case of the quenched APZ sample, at *T* = 363 K the X-ray diffraction pattern revealed the crystallization of form III, which partially converted into a mixture of forms IV and form I for *T* > 400 K. The presence of form I, which was not registered in DSC curves, could be due to different heating rates applied by these two techniques (0.3 K/min in XRD vs 10 K/min in DSC). The situation was quite different for APZ incorporated into silanized silica templates. In such a case, it should be noted that the main contribution to the diffraction pattern, visible as a broad peak with a maximum at around 20°, came from the amorphous structure of silica. The amount of APZ incorporated into the silica template was small and it gave only a very weak contribution to the XRD data. However, it was possible to derive some reliable observations on the recrystallization of APZ from the supercooled liquid phase during heating inside the pores. First, the XRD data indicated the crystallization of a mixture of forms I and III from the quenched API inside of the silanized SiO_2_ pores. On further heating, around *T* ∼ 413 K, form III was melted, and at *T* > 423 K, the sample was already fully amorphous after the melting of form I. Therefore, the analysis of temperature-dependent X-ray diffraction patterns suggests that in silanized silica pores actually I (higher *T*_m_) and III (lower *T*_m_) polymorphs are formed, not III and IV, as deduced from DSC studies. This apparent inconsistency is fully understandable. To explain that one should emphasize that the identification of the given polymorph from the analysis of the melting temperature is not reliable, since *T*_m_ generally decreases with the pore diameter, as predicted by the Gibbs–Thomson relationship. In such a case, the more accurate are conclusions derived from XRD investigations. Therefore, we may state that in silanized SiO_2_ pores, in fact, polymorphs I and III are formed during the heating of the confined samples. For APZ confined within AAO templates, a behavior similar to that detected for silanized SiO_2_ was found. As can be seen in Figure S2, presenting the evolution of the XRD data with temperature for APZ in alumina pores, the crystallization of polymorphic forms I and III is visible as well. The order of crystallization of both polymorphs in these membranes seems to be reversed compared to that of silanized SiO_2_. Furthermore, considering that (i) the pore radius of native and silanized silica membranes is comparable and (ii) melting temperatures of APZ infiltrated into these membranes are almost the same, one can expect that the same polymorphs (I and III) of APZ are formed in native SiO_2_. Bearing in mind all the above deliberations, one can rule out the formation of different polymorphs as an explanation of much different crystallization patterns of APZ confined within native (two-step crystallization) and silanized (one-step crystallization) silica templates during heating with different rates. One can suppose that the former phenomenon might be in fact related to the solidification of the interfacial and core molecules, which is most likely prevented in the silanized SiO_2_ pores since, in that case, intermolecular interactions between host and guest molecules must be changed.

**Figure 4:**
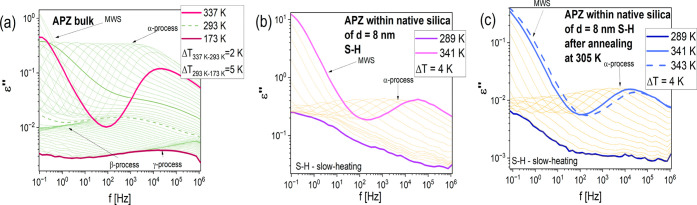
Dielectric loss spectra of bulk APZ (a) and the sample infiltrated into native SiO_2_ templates collected on “slow-heating” protocol (b). Panel (c) presents the dielectric loss spectra of APZ within native SiO_2_ templates after the annealing procedure.

Having obtained the results of DSC and XRD experiments, we carried out molecular dynamics studies using the BDS method. Note that, as mentioned in the “Materials and Methods” section, for the freshly prepared confined samples, two different thermal protocols, labeled respectively (i) “slow-cooling (S–C) and (ii) “slow-heating” (S–H), of the beforehand quenched sample were applied during dielectric measurements. The loss spectra collected above and below *T*_g_ for bulk APZ are presented in Figure 4(a). In turn, Figure 4(b) shows the analogous data obtained for the API infiltrated into native SiO_2_ (measured according to the S–H procedure). Additionally, the representative dielectric loss spectra of APZ within two other types of membranes (measured according to the S–C protocol) have been presented in Figure S3. As can be observed, in the case of bulk and infiltrated samples, there is one well-resolved structural (α) relaxation in the collected spectra, related to the cooperative motions of molecules, which is preceded either by the direct current (dc) conductivity, related to the charge transport of ionic moieties (bulk APZ) or the Maxwell–Wagner–Sillars (MWS) interfacial polarization (confined systems). Note that the MSW polarization appears also in the spectra of bulk APZ measured at higher *T* (>333 K), where the sample starts to crystallize. Both, α- and dc conductivity/MWS processes move toward lower frequencies with decreasing temperature. In the case of the bulk sample, the amplitude of the α-relaxation peak decreases rapidly at higher *T* (Figure 4(a)) indicating the occurrence of the crystallization process. Such a situation is commonly observed for many APIs.^33,64,65^ On the other hand, this phenomenon was much less pronounced for the API infiltrated into native SiO_2_ (Figure 4(b)) and other confined systems, regardless of the applied measurement protocol. This suggests a much lower degree of crystallization of APZ in mesopores, with respect to the bulk sample at higher temperatures. However, it should be emphasized that during BDS measurements, we did not reach the temperatures at which the crystallization occurred (compared to calorimetric measurements). Therefore, it cannot be excluded that the crystallization would have occurred if we had performed dielectric measurements experiments above *T* > 343 K. One can also mention that dielectric spectra of bulk APZ measured below *T*_g_ (Figure 4(a)) showed the presence of two secondary relaxations (β and γ), faster than the α-process, whose molecular origin will be clarified later on (the β-process was also visible at *T* > *T*_g_). Simultaneously, these relaxations were not observed in the loss spectra of the confined samples. It is worth adding that, as for cilnidipine,^34^ which has been recently studied by us, in the case of nanospatially restricted APZ, dielectric spectra do not reveal any sign of the “interfacial process” related to the reorientational motions of the molecules adsorbed at the surface of the pore walls. A similar scenario has been reported in the literature for many confined glass formers.^46,66−69^ It is not a surprising finding since the occurrence of this mode depends on various factors, like equilibrium time scales of the experiment or mass exchange between two layers of molecules inside the pores.^70,71^

**Figure 5:**
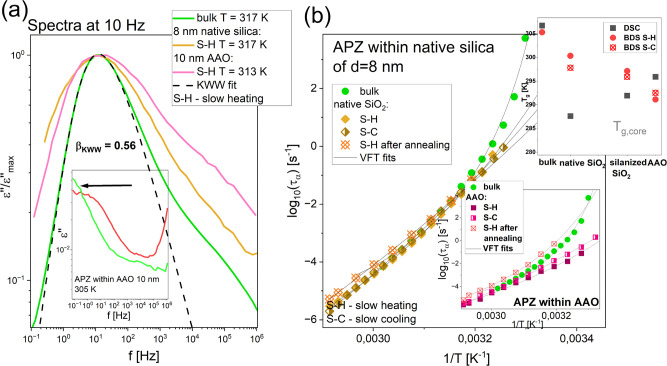
Comparison of the representative normalized loss spectra measured for bulk APZ and confined samples at 10 Hz (a). The dashed line represents the KWW fit. Temperature dependences of the α-relaxation times for bulk APZ and API infiltrated into native SiO_2_ templates (b). The lower inset in panel (b) presents the same dependencies for APZ confined within AAO membranes. Solid lines are the best VFT fits (eq 4). In turn, the upper inset in panel (b) shows the values of *T*_g,core_ determined from DSC and BDS data for bulk and confined systems.

Subsequently, in Figure 5(a), we compared the shape of the structural (α)-relaxation process for bulk APZ, as well as the API incorporated into native SiO_2_ and AAO systems, collected during the “slow-heating” procedure. To do that, dielectric loss spectra with a maximum of the α-peak at around 10 Hz (collected at similar *T* conditions), were chosen, subsequently normalized with respect to the maximum of dielectric loss (ε”_max_), and superposed. Next, the α-loss peak for the bulk APZ was fitted to the one-sided Fourier transform of the Kohlrausch–Williams–Watts (KWW) function (the dashed line in Figure 5(a)):^72,73^
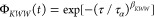
2in which the fractional exponent, *β*_*KWW*_, describes the α-relaxation time distribution’s nonexponentiality. The obtained value of this parameter was equal to 0.56. In comparison to the bulk sample, the α-peaks of both confined systems are wider. It is an often observed phenomenon, related to the increasing heterogeneity of relaxation dynamics under confinement, due to interactions between the sample and pore walls.^74,75^

In the next step, dielectric loss spectra collected for APZ infiltrated into alumina and two kinds of silica templates (measured using two different thermal protocols described in the “Materials and Methods” section) were analyzed using the superposition of two Havriliak–Negami (HN) functions:^76^
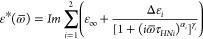
3where ε_∞_ is the high-*f* limit permittivity, Δε is the dielectric relaxation strength, ω̅ is the angular frequency (ω̅ = 2*πf*), α and γ are the parameters, which characterize the shape of a given relaxation peak, and *τ*_*HN*_ is the HN relaxation time. In the case of the bulk APZ, to fit the experimental data, one HN function with the additional conductivity term (, where σ_dc_ is the dc conductivity) was applied. Then, using the approach described in ref (77), structural relaxation times (*τ*_*α*_) have been calculated from *τ*_*HN*_, and plotted as a function of inverse temperature in Figures 5(b) and S4. To determine the values of the glass transition temperature (*T*_g_) of all examined systems (*T*_g,core_ in the case of confined samples), *τ*_*α*_(*T*)-dependences were fitted to the Vogel–Fulcher–Tammann (VFT) equation (solid lines in Figure 5(b) and its downer inset, as well as in Figure S4):^78−80^
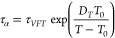
4where *τ*_*VFT*_ is the relaxation time at finite *T*, *D*_*T*_ is the strength parameter, and *T*_0_ corresponds to *T* at which *τ*_*α*_ → ∞. In our studies, *T*_g_ was defined as a *T* at which *τ*_*α*_ is equal to 100 s (log_10_*τ*_*α*_ = 2).^77,81^ It should be added that for bulk APZ the temperature dependences of secondary relaxation processes (β and γ) and their molecular origin were also examined. We determined the following values of the activation energy: *E*_*β*_ = 79.9 kJ/mol and *Eγ* = 29.5 kJ/mol (*T* < *T*_g_). Moreover, based on the coupling model (CM) predictions,^82^ we concluded that the β-relaxation is probably a Johari–Goldstein (JG) process, which originates from local, noncooperative motions of the whole APZ molecules, while the source of the γ-mode are rotations of polar parts of API molecules (it is a non-JG relaxation of intramolecular character). The results and description of the mentioned analyses are presented in the SI.

Interestingly, as can be seen in Figure 5(b), there is a characteristic change in the slope of τ_α_(*T*) for both confined APZ systems measured directly after preparation (regardless of the measurement protocol), with respect to the bulk sample (the same situation was found for APZ in silanized silica pores, see Figure S4). It is a quite universal pattern of behavior of the low- and high-molecular-weight systems infiltrated into porous media. This phenomenon is widely discussed in the context of the free volume,^68,83^ dynamic heterogeneity,^84,85^ and spinodal temperature^86,87^ concepts, or it is assigned to the vitrification of the interfacial layer.^88,89^ As a consequence of that, confined systems can be considered as being in quasi-isochoric conditions at lower temperature and structural/segmental relaxation times follow characteristic *τ*_*α*_(*T*) at constant volume.

Having at hand dielectric and calorimetric data, we compared the *T*_g_s determined from both experimental techniques for the bulk and confined sample (please see the upper inset in Figure 5(b)). For the latter ones, *T*_*g*_s from both measurement protocols (S–H and S–C), which differed only slightly, were presented. In the case of bulk APZ, the agreement between dielectric and calorimetric values (*T*_g_^BDS^ and *T*_g_^DSC^) is very good. On the other hand, we found some discrepancies between low *T*_g_s in DSC thermograms and those determined from BDS data (S–H and S–C procedures) for the confined systems (*T*_g_,_core_s). However, these differences, except for those observed for APZ in native SiO_2_ pores, are not significant and are due to the varying heating and cooling rates applied in both experiments.

As a further point of dielectric investigations, we performed annealing of the API infiltrated into silica and alumina pores at *T* = 305 K (located below *T*_g,interfacial_, and above *T*_g,core_ determined from DSC studies) that lasted for a month. The main aims of these experiments were to (i) check the physical stability of the API in porous templates over a longer time and (ii) verify whether confinement effects can be erased (short-term annealing ∼ 250 h). The representative data collected during these experiments are shown in the lower inset of Figure 5(a). Time-dependent measurements revealed that the position of the structural process in the collected spectra changes (they shift to lower frequencies). Further temperature-dependent experiments on the fully annealed sample (incorporated into native silica and alumina pores Figure 4(c)) indicated that *τ*_*α*_(*T*) dependences follow bulk-like behavior; see Figure 5(b). Hence, all confinement effects were completely erased after 250 h annealing at *T*_anneal_ = 305 K. For APZ within silanized SiO_2_ pores (Figure S4), a similar effect was not detected probably due to modification of interactions between the API and the porous matrix. The observation shown in the case of the two examined systems agrees with the recent papers showing that for the confined materials prepared as thin films or incorporated into porous media, annealing experiments allow for recovering bulk-like dynamics. The other benefit of long-time dielectric experiments was to check whether there is any trace of crystallization of API infiltrated in pores around room temperature (experiments that lasted for a month). Herein, it is important to note that due to the high tendency to crystallization of this API during heating in DSC experiments, this technique was not suitable to address this problem in a proper way. Moreover, XRD investigations were not so sensitive to determine the traces of crystallization in confined API stored at room temperature due to strong matrix diffraction and a low degree of API crystallization.

The analysis of dielectric data obtained during and after prolonged annealing indicated that APZ did not crystallize over one month of storage at around room temperature. The only effect we observed is the increase in the amplitude of the structural process (for the crystallization process, the opposite effect should be observed), which was accompanied by the recovering bulk-like dynamics. Therefore, it can be concluded that mesoporous systems as specific drug carriers could protect amorphous APZ against recrystallization.

**Figure 6:**
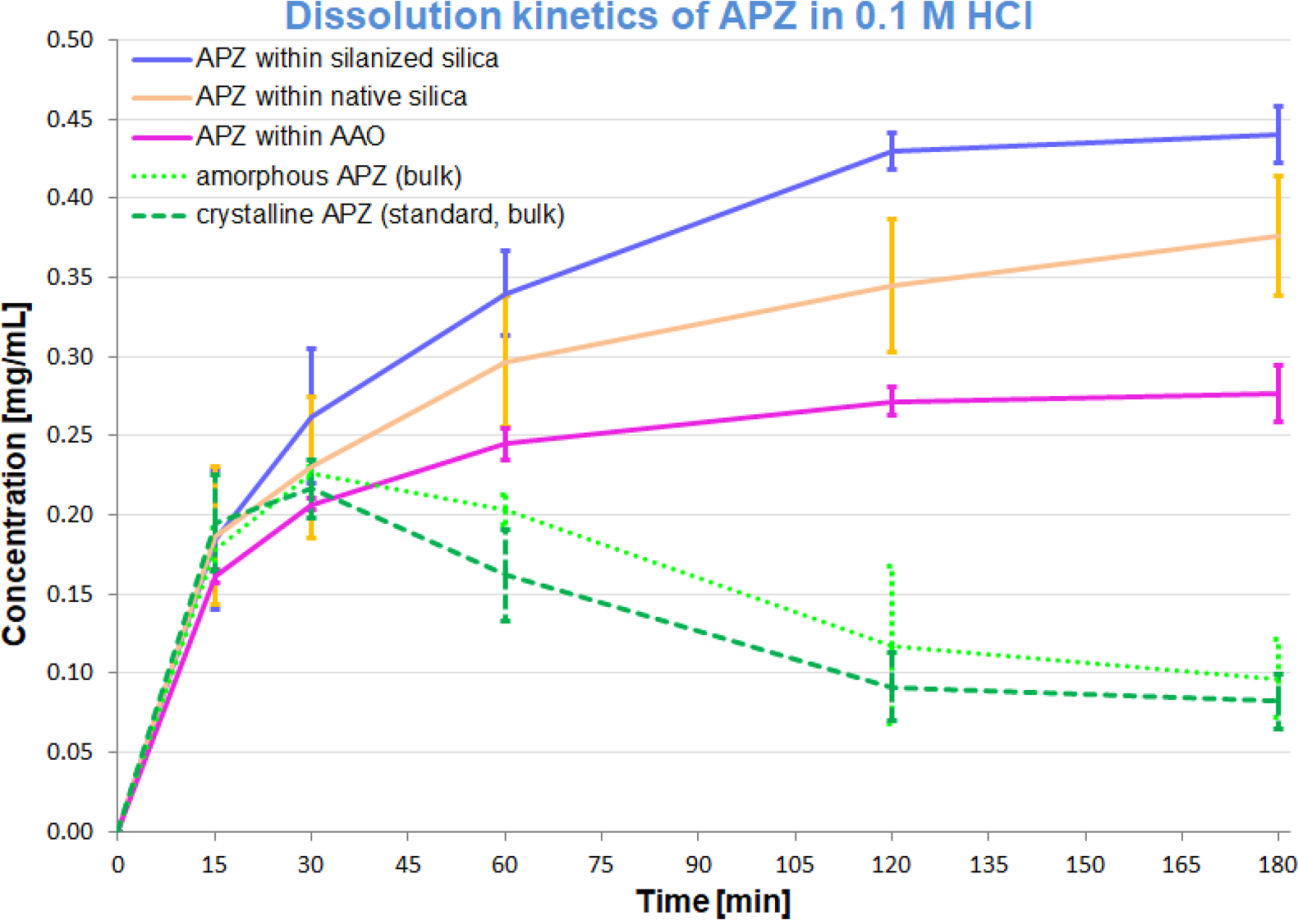
Rate of dissolution of crystalline and amorphous APZ, as well as API incorporated into AOO, native, and silanized silica matrices in 0.1 M HCl.

In the final part of our studies, we investigated the dissolution rates of crystalline and amorphous APZ, as well as API incorporated into three mesoporous matrices (native SiO_2_, silanized SiO_2_, and AAO). The results are listed in Figure 6. As can be seen, the concentration of dissolved crystalline and amorphous API in an acidic medium reaches about 0.2 mg/mL within the first 30 min and then gradually decreases to a stable low level (about 0.1 mg/mL) after about 2 h. The solubility of the amorphous form is slightly higher than the crystalline one, but these differences are negligible. This may be due to local supersaturation of the solution, which triggers rapid precipitation of the crystalline form with lower solubility. A completely different situation is observed for APZ incorporated into mesoporous templates. In all cases, a steady, slow increase in APZ concentration is visible without signs of precipitation. Hence, herein, the so-called parachute effect is prolonged, i.e., the substance is released gradually and effectively dispersed in the entire solution. It is in contrast to the crystalline and amorphous samples, where the mentioned effect is definitely shorter probably due to local supersaturation, which induces the recrystallization of the drug substance.

The greatest improvement in solubility occurred for APZ infiltrated into silanized SiO_2_ pores, while the least was observed for API incorporated into AAO matrices. Differences between the templates are most likely the results of various properties of each material, e.g., surface wettability, and thus different types or strengths of API-matrix interactions. The probable explanation for the higher dissolution rate of APZ incorporated into silanized silica with respect to the native silica template is the fact that the specific interactions (H-bonds) between the porous membrane and the API molecules are weakened due to silanization (as a result of this process, the density of silanol groups is lowered, and the surface wettability changes). Importantly, in all confined systems the solubility was significantly greater than for the bulk (crystalline and amorphous) and the dissolution curves were close to the desired so-called extended release pattern (logarithmic increase in API concentration until a stable maximum is reached). Hence, one can assume that the use of mesoporous matrices as novel drug carriers appears to be an interesting perspective that can improve the dissolution of APZ and other poorly soluble APIs from oral solid dosage forms. Furthermore, by increasing the concentration at the absorption site, mesoporous carriers may increase the bioavailability of these APIs. In addition, the prolonged release effect may contribute to a more convenient dosage regime (reduced frequency of drug administration, fewer fluctuations of drug concentration in blood, etc.).

## Conclusions

In this work, we performed calorimetric, X-ray diffraction, dielectric, and dissolution studies on the antipsychotic API, aripiprazole infiltrated into AAO (*d* = 10 nm), as well as native and silanized SiO_2_ (*d* = 8 nm) mesoporous matrices. Standard and nonisothermal DSC investigations suggested the recrystallization of amorphous API to polymorphic forms IV and III both in bulk and in each confined system. Nevertheless, more reliable temperature-dependent XRD experiments indicated that, in fact, during heating, amorphous confined APZ recrystallized to the same polymorphic forms as those occurring in the initial crystalline sample (i.e., III and I). In the case of amorphous bulk API, the formation of polymorph III, which at higher *T* converted into the mixture of forms IV and I, was observed. One can also mention that nonisothermal calorimetric data of APZ infiltrated within native SiO_2_ pores showed a double crystallization peak in thermograms at lower heating rates, which was not observed in the case of silanized SiO_2_ membranes (one crystallization peak). We suggested that the reason for a two-step crystallization process in the former sample could be the solidification of the interfacial and core molecules. Additionally, dielectric measurements of three examined confined systems revealed that at some temperature, corresponding to the vitrification of interfacial molecules, there is a change in the molecular dynamics with respect to the bulk sample. Interestingly, after a short-time annealing (ca. 250 h) at *T* = 305 K, the confined effects in two examined systems were erased. Consequently, the structural dynamics of APZ incorporated into native silica and alumina mesoporous membranes showed a bulk-like behavior. Based on the outcomes of long-term annealing measurements, we deduced that the amorphous APZ incorporated into porous templates remains stable during 1 month of storage around room temperature. Finally, dissolution rate measurements revealed that in contrast to crystalline and bulk samples, infiltrated APZ reveals a logarithmic increase in API concentration in the used 0.1 M HCl solution over time (similar to a prolonged release effect) without any sign of precipitation. All obtained results suggest that mesoporous membranes can be used in the future as potential carriers for APZ and other easily recrystallizing APIs, providing not only stability but also the desired release profile in the blood.

## Supporting Information Available

Detailed procedures of confined samples’ preparation, temperatures of glass transition, crystallization, and melting, determined from nonisothermal DSC measurements for bulk APZ and APZ incorporated into various porous membranes, Kissinger plot for the API incorporated into native silica templates, evolution of XRD patterns on heating of APZ in AAO pores, temperature dependences of α-relaxation times for bulk APZ and API infiltrated into silanized SiO_2_ membranes, relaxation maps of the bulk APZ together with the description of the calculation of activation energy for both secondary processes (β, γ), as well as determination of primitive relaxation times (τ_0_) using the Coupling Model (PDF)
