# Supplementary material for: Mesoporous Matrices as a Promising New Generation of Carriers for Multipolymorphic Active Pharmaceutical Ingredient Aripiprazole
Source: Mol Pharm. 2023 Sep 27;20(11):5655–67. doi: 10.1021/acs.molpharmaceut.3c00524 (PMC10630940; doi:10.1021/acs.molpharmaceut.3c00524)
Supplement: Supplementary file 1 — mp3c00524_si_001.pdf [file mp3c00524_si_001.pdf]

Supporting Information

Mesoporous matrices as promising new generation of carriers for multi-polymorphic active pharmaceutical ingredient - aripiprazole

Aldona Minecka,[a,*] Magdalena Tarnacka,[b] Karolina Jurkiewicz[b],
Daniel Żakowiecki,[c] Kamil Kamiński,[b] Ewa Kamińska[a,*]

[a] *Department of Pharmacognosy and Phytochemistry, Faculty of Pharmaceutical Sciences in Sosnowiec, Medical University of Silesia in Katowice, 41-200 Sosnowiec, Poland*

[b]*A. Chelkowski Institute of Physics, University of Silesia in Katowice, 41-500 Chorzow, Poland;*

[c] *Chemische Fabrik Budenheim KG, Rheinstrasse 27, 55257 Budenheim, Germany*

*Corresponding author: aldona.minecka@sum.edu.pl; ekaminska@sum.edu.pl

Table of content

Detailed procedures of the confined samples' preparation 2

Table S1. Values of T_g T_c, T_{m1}, and T_{m2} determined from non-isothermal DSC measurements for bulk APZ. 2

Table S2. Values of both T_g ($T_{g,core}$, $T_{g,interfacial}$), T_c, T_{m1}, and T_{m2} determined from non-isothermal DSC measurements for APZ within native silica of d = 8 nm. 3

Table S3. Values of both T_g ($T_{g,core}$, $T_{g,interfacial}$),T_c, T_{m1}, and T_{m2} determined from non-isothermal DSC measurements for APZ within silanized silica of d = 8 nm. 3

Table S4. Values of T_g ($T_{g,core}$, $T_{g,interfacial}$),T_c, T_{m1}, and T_{m2} determined from non-isothermal DSC measurements for APZ within AAO of d = 10 nm. 3

Fig. S1. Kissinger plot for exothermic crystallization peaks observed for APZ incorporated into native silica templates. 4

Fig. S2. Evolution of XRD patterns on heating of APZ in AAO pores 4

Fig. S3. Representative dielectric loss spectra of APZ infiltrated into AAO membranes with d = 10 nm (a) and silanized silica templates with d = 8 nm (b) measured on "slow-cooling" (S-C) protocol. 5

Fig. S4. Temperature dependences of α-relaxation times for bulk APZ and API infiltrated into silanized SiO_2 templates. Solid lines represent VFT fits. 5

Fig. S5. Relaxation map of bulk APZ. The open stars are the primitive relaxation times (τ_0) of the CM calculated with n=0.44 (β_{KWW}=0.56) at several temperatures close to T_g from the corresponding experimental τ_α and values given by the VFT fit. 6

Detailed procedures of the confined samples' preparation

Firstly, all types of membranes were dried in an oven at T = 423 K under vacuum (10^{-2} bar) for t = 24 h to remove any volatile impurities from the nanochannels before filling. Next, the cooled and weighed unfilled membranes were top-coated with the crystalline APZ. Subsequently, the whole system was kept at T = 420 K for at least 4-5 h, until the mass of membranes increased to constant values. During this time, the melted API flowed inside the nanochannels by infiltration forces. After completing this process, the surface of all membranes was heated again up to T = 420 K at 0.1 MPa to remove the excess sample on the surface with a metal blade and a paper dust-free wipe.

Table S1. Values of T_g T_c, T_{m1}, and T_{m2} determined from non-isothermal DSC measurements for bulk APZ.

ϕ [K/min]	T_g [K] ± 1K	T_c [K] ± 1K	T_{m1} [K] ± 1K	T_{m2} [K] ± 1K
2,5	305.9	357.9	403.9	412.1
5	306.2	358.1	404.7	412.2
7.5	306.4	361.9	405.6	412.8
9	306.5	362.6	406.2	412.9
10	306.7	363.4	406.7	413.1
12.5	307.1	365.3	407.1	412.8
15	307.3	368.6	407.5	412.4
20	307.6	372.1	407.7	411.5
25	307.8	371.9	407.9	411.9

Table S2. Values of both T_g ($T_{g,core}$, $T_{g,interfacial}$), T_c, T_{m1}, and T_{m2} determined from non-isothermal DSC measurements for APZ within native silica of d = 8 nm.

ϕ [K/min]	$T_{g,core}$ [K] ± 1K	$T_{g,interfacial}$ [K] ± 1K	T_{c1} [K] ± 1K	T_{c2} [K] ± 1K	T_{m1} [K] ± 1K	T_{m2} [K] ± 1K
2.5	280.3	333.3	344.3	353.0	405.3	410.4
5	285.1	336.6	351.9	359.6	406.7	411.2
6	285.1	336.9	354.8	362.2	406.5	411.1
7.5	285.3	338.7	358.6	363.9	406.1	411.2
10	287.6	342.4	--	363.9	406.1	411.2
12.5	285.6	345.3	--	368.6	405.4	410.1
15	286.7	346.0	--	368.4	406.1	409.8
17.5	286.2	346.9	--	372.1	405.4	--
20	286.9	346.8	--	370.5	406.4	--

Table S3. Values of both T_g ($T_{g,core}$, $T_{g,interfacial}$),T_c, T_{m1}, and T_{m2} determined from non-isothermal DSC measurements for APZ within silanized silica of d = 8 nm.

ϕ [K/min]	$T_{g,core}$ [K] ± 1K	$T_{g,interfacial}$ [K] ± 1K	T_c [K] ± 1K	T_{m1} [K] ± 1K	T_{m2} [K] ± 1K
5	289.1	322.9	362.4	405.7	410.3
7.5	288.4	326.0	369.2	405.7	410.1
10	291.9	330.3	370.5	405.6	410
12.5	290.5	329.4	376.2	405.5	--
15	291.6	331.6	378.0	405.4	--
17.5	291.7	331.3	381.0	405.1	--

Table S4. Values of T_g ($T_{g,core}$, $T_{g,interfacial}$),T_c, T_{m1}, and T_{m2} determined from non-isothermal DSC measurements for APZ within AAO of d = 10 nm.

ϕ [K/min]	$T_{g,core}$ [K] ± 1K	$T_{g,interfacial}$ [K] ± 1K	T_c [K] ± 1K	T_{m1} [K] ± 1K	T_{m2} [K] ± 1K
7.5	295.2	329.4	357.5	405.7	410.5
10	295.9	330.1	360.8	404.9	410
15	296.1	331.6	362.8	405.4	409.9
20	296.6	333.1	369.0	405.7	411.2

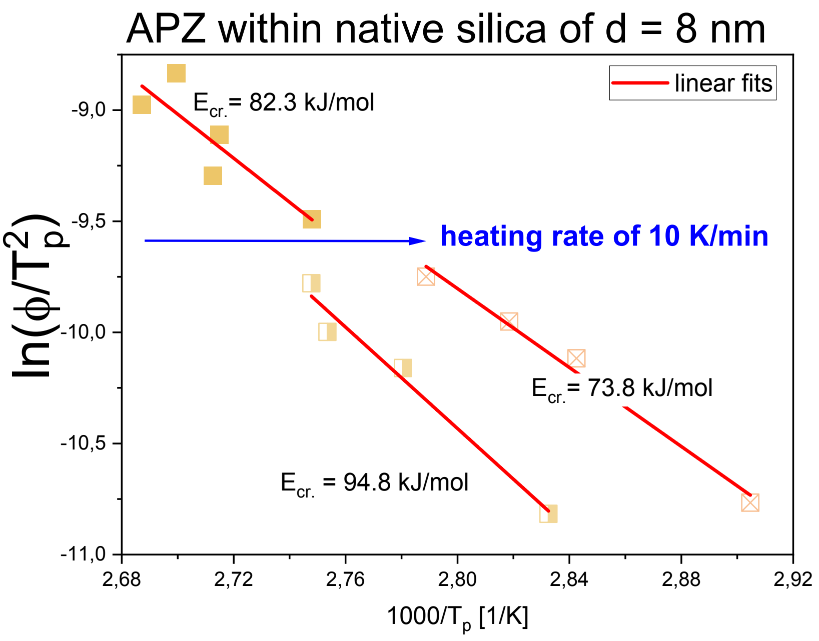

Fig. S1. Kissinger plot for exothermic crystallization peaks observed for APZ incorporated into native silica templates.

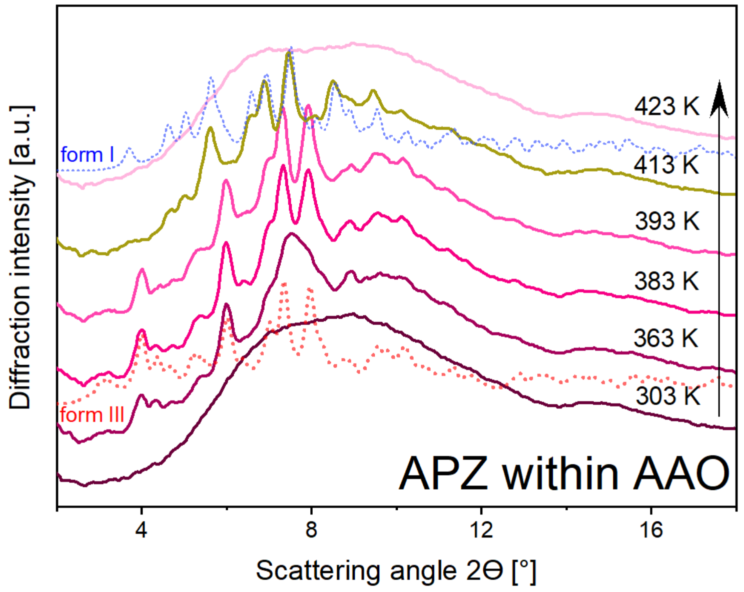

Fig. S2. Evolution of XRD patterns on heating of APZ in AAO pores.

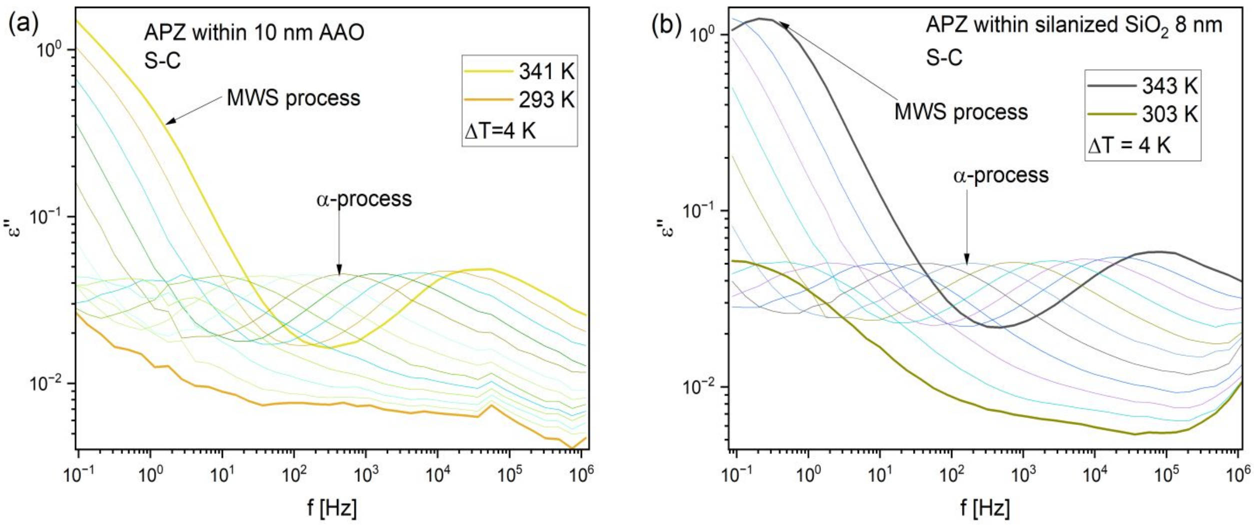

Fig. S3. Representative dielectric loss spectra of APZ infiltrated into AAO membranes with *d* = 10 nm (a) and silanized silica templates with *d* = 8 nm (b) measured on "slow-cooling" (S-C) protocol.

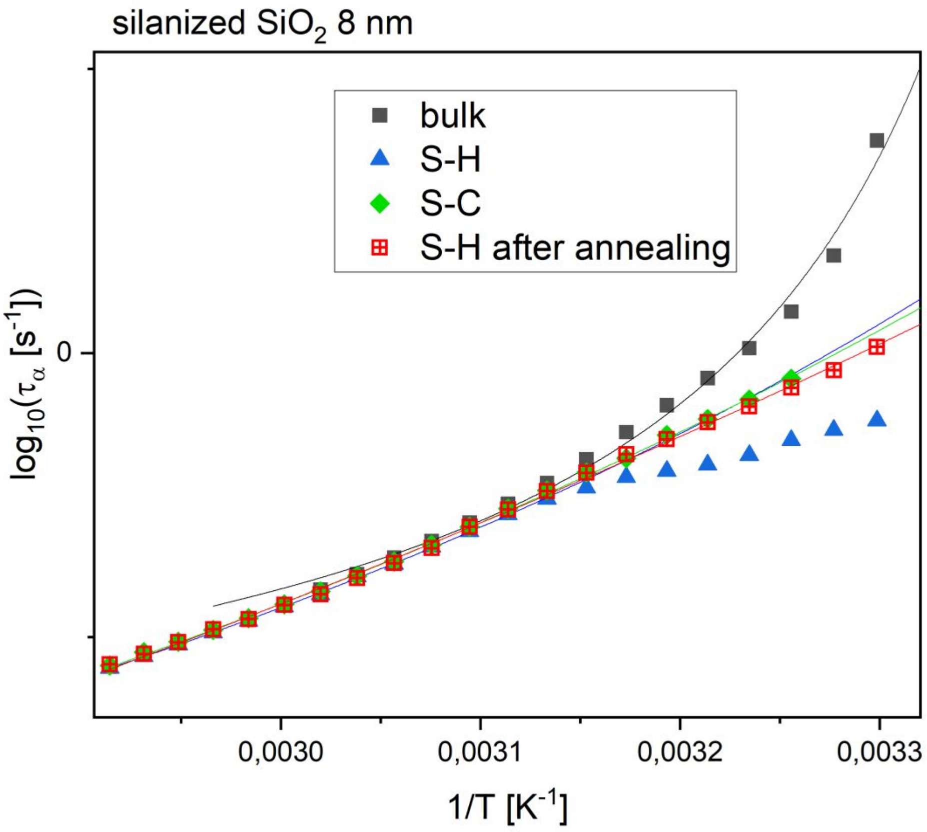

Fig. S4. Temperature dependences of α-relaxation times for bulk APZ and API infiltrated into silanized SiO_2 templates. Solid lines represent VFT fits.

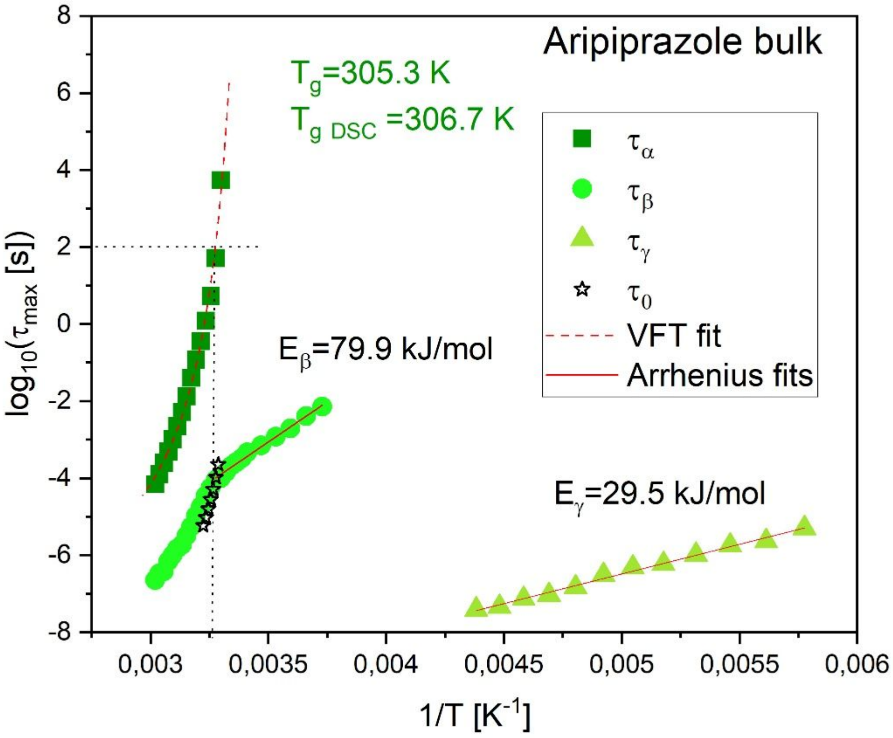

Fig. S5. Relaxation map of bulk APZ. The open stars are the primitive relaxation times (τ_0) of the CM calculated with n=0.44 (β_{KWW}=0.56) at several temperatures close to T_g from the corresponding experimental τ_α and values given by the VFT fit.

In the case of secondary relaxation processes, the Arrhenius power law was used to fit the temperature dependences of τ_β and τ_γ (solid red lines in Fig. S5), and consequently, determine the activation barrier (E_x, where $x = \beta, \gamma$):

$$\tau = \tau_\infty exp\left(\frac{E_x}{RT}\right), \quad \text{(S1)}$$

where τ_∞ is the pre-exponential factor, while R is the gas constant.

Moreover, in order to determine the molecular origin of both secondary processes in APZ, especially check, which one is the true Johari-Goldstein (JG) relaxation of intermolecular origin, we applied the Coupling Model (CM) by Ngai.[1,2] This approach assumes the following relation between the JG relaxation time (τ_{JG}) and the primitive relaxation time (τ_0) of the CM:

$$\tau_{JG}\,(T, p) \approx \tau_0\,(T, p) \quad \text{(S2)}$$

The value of τ_0 can be determined from the parameters τ_α and the stretched exponent of the KWW function ($\beta_{KWW} = 1 - n$, where n is a coupling parameter) by the formula:

$$\tau_0 = (t_c)^n(\tau_\alpha)^{1-n} \tag{S3}$$

t_c is a constant, which is equal to 2 ps for most polymeric and low-molecular-weight glass formers.[1,3] We calculated τ_0 (open stars in Fig. S5) at several temperatures close to T_g from the corresponding $\tau_\alpha(T)$ of APZ and found that they are close to experimental β-relaxation times. Hence, the good correspondence between τ_0 and τ_β indicates that the β-mode is a true JG-process, whose source are local, non-cooperative motions of the entire API molecules. In turn, the faster γ-relaxation is a non-JG process of intramolecular character, which probably originates from the rotations of polar groups of APZ molecule.

References

(1) Ngai, K. L.; Paluch, M. Classification of Secondary Relaxation in Glass-Formers Based on Dynamic Properties. *J. Chem. Phys.* **2004**, *120* (2), 857-873. https://doi.org/10.1063/1.1630295.

(2) Ngai, K. L. An Extended Coupling Model Description of the Evolution of Dynamics with Time in Supercooled Liquids and Ionic Conductors. *J. Phys.: Condens. Matter* **2003**, *15* (11), S1107. https://doi.org/10.1088/0953-8984/15/11/332.

(3) Ngai, K. L. Coupling Model Explanation of Salient Dynamic Properties of Glass-Forming Substances. *IEEE Trans. Dielectr. Electr. Insul.* **2001**, *8* (3), 329-344. https://doi.org/10.1109/94.933340.
